# Navigating Structural Tensions: Australian Surrogacy Facilitators’ Understanding on Children’s Rights in Cross-Border Surrogacy

**DOI:** 10.1007/s41649-025-00364-2

**Published:** 2025-09-15

**Authors:** Yingyi Luo

**Affiliations:** Melbourne Law School, University of Melbourne, Melbourne, Australia

**Keywords:** Children’s rights, Cross-border surrogacy, Surrogacy facilitators, Australia, Law

## Abstract

Within cross-border surrogacy, particularly in consumer countries, the protection of children’s human rights presents complex and significant challenges. This study examines how Australian surrogacy facilitators perceive and navigate the rights of children born through such arrangements. Despite striving to adopt child-centred practices, facilitators face structural barriers, including conflicting interests between intended parents and children, the commodification of children in surrogacy contracts, and legal ambiguities around parental relationships. These challenges create a persistent tension between the universal recognition of children as independent rights holders and the practical demands of facilitating surrogacy arrangements. The findings highlight the need for robust regulatory frameworks and clearer accountability mechanisms to ensure children’s rights are upheld. This study advances the scholarship on surrogacy and human rights and informs the ongoing review of Australian surrogacy laws, emphasizing the need for stronger protections for children in international surrogacy arrangements.

## Introduction

Commercial surrogacy frequently involves profit-driven entities, such as agencies and brokers, that arrange, negotiate, and manage surrogacy agreements for financial gain (Horsey 2023). These arrangements typically occur within the framework of assisted reproductive technologies, including in vitro fertilization and embryo transfer for gestational surrogacy—where the surrogate mother has no genetic link to the child—and artificial insemination for traditional surrogacy, where the surrogate mother is genetically related to the child (Payne et al. 2020; Ellenbogen et al. 2021; United Nations 2018). Regulatory frameworks categorize surrogacy into two distinct types: altruistic surrogacy, where the surrogate mother receives no financial compensation beyond reimbursement for medical and related expenses, and commercial surrogacy, where financial remuneration is provided for the surrogate’s services (Stuhmcke 1996). This distinction has partly contributed to the rise of cross-border surrogacy, as intended parents often turn to countries where commercial surrogacy is legal or less regulated to circumvent restrictions or high costs in their home countries (Zannettino et al. 2019; Hibino 2022).

Australia’s restrictive surrogacy laws have made it one of the largest exporters of intended parents worldwide, prompting serious concerns about the protection of children’s rights in international surrogacy arrangements (Zannettino et al. 2019; Everingham and Whittaker 2023; Kneebone et al. 2023). High-profile cases, such as the Baby Gammy incident, highlight the significant vulnerabilities faced by children born through cross-border surrogacy. In this case, an Australian couple commissioned a Thai surrogate who gave birth to twins; the couple returned to Australia with the healthy child, leaving behind Gammy, who was born with Down syndrome (ABC News 2016). This situation drew Australia’s attention to the ethical and human rights concerns inherent in international surrogacy arrangements. In response, a 2016 Australian government inquiry stated that “Australians who broker, facilitate or engage in offshore surrogacy arrangements (should be) aware of the human rights risks those arrangements may pose” (Standing Committee on Social Policy and Legal Affairs 2016, 32). Building on this earlier inquiry, in 2024, the Albanese Government requested the Australian Law Reform Commission (AL RC) to conduct a comprehensive review of Australian surrogacy laws to ensure that legal frameworks protect the rights of children born through surrogacy (Australian Law Reform Commission 2024).

Against this background, this paper explores how Australian surrogacy facilitators, working with intended parents pursuing cross-border surrogacy, perceive their professional responsibilities through a children’s rights perspective. For the purposes of this paper, surrogacy facilitators are defined as individuals providing information and services related to cross-border surrogacy, including consumer advocates, lawyers, and counselors. It is a fundamental legal principle in Australia that all children, including those born through surrogacy arrangements, have a broad set of rights as formulated in the United Nations Convention on the Rights of the Child (hereinafter referred to as the CRC) (Human Rights Law Centre 2015). This includes respecting children as autonomous subjects of rights rather than merely beneficiaries of adult benevolence (CRC General Comment No. 13, para. 72a).

However, the characterization of commercial surrogacy as akin to “selling babies” or “human trafficking” highlights the tension between commodification and human rights (United Nations 2018). Predominantly intended parent-centred and profit-driven market practices have been identified as neglectful of children’s rights, creating barriers to the full realization and enjoyment of those rights (United Nations 2018; Fronek 2018). The rights discourse in cross-border surrogacy is fraught with conflicting interests, particularly between the intended parents’ right to parenthood and the child’s best interests, which are not always mutually supportive (Achmad 2022). The lack of agency due to their infancy and the absence of independent representation exacerbate the vulnerability of these children, potentially compromising their interests when they conflict with those of the adults involved (Wade 2017). While facilitators are key actors in managing the complex dynamics of cross-border surrogacy and third-party-assisted reproduction arrangements—bridging legal and informational gaps (Luo et al. 2023; Millbank 2015a, b; Shenfield et al. 2011)—their involvement can significantly impact the extent to which children born through these arrangements are recognized and treated as independent rights holders. This article contributes to the scholarship on surrogacy and human rights by analyzing the complex role of surrogacy facilitators and the challenges they face in upholding children’s rights in cross-border surrogacy. The aim of this paper is to explore how these professionals perceive and value the rights of children in their work and how they balance this understanding with normative and practical considerations. This exploration can directly contribute to the review of Australian surrogacy law by highlighting the need for clearer accountability frameworks, ensuring stakeholders uphold children’s rights as autonomous rights holders in cross-border surrogacy arrangements.

This paper begins by presenting the current literature on cross-border surrogacy, with a particular focus on children’s rights and the role of market actors. The subsequent section situates the research within the Australian context by providing an overview of its legal framework governing both domestic and international surrogacy arrangements. It then presents the empirical data from the study, detailing the methods of data collection and analysis. The results section describes how surrogacy facilitators articulate the meaning of children’s rights and navigate various considerations in their professional roles within cross-border surrogacy arrangements. Finally, the discussion interprets the interview findings with surrogacy facilitators, linking them to the broader literature on cross-border surrogacy facilitation and highlighting how structural constraints impact facilitators’ ability to uphold children’s rights and addresses the implications of these findings for the future regulation, emphasizing the need for a framework that ensures the protection and prioritization of children’s human rights in cross-border surrogacy arrangements.

## Research Background: Cross-Border Surrogacy, Children’s Rights, and the Role of Market Actors

Cross-border surrogacy is a highly contested practice that exacerbates gender, class, and racial inequalities by commodifying the reproductive labour of women in less-developed countries while enabling wealthier individuals from developed nations to pursue parenthood through outsourced reproductive services (Kirby 2014; Deomampo 2016; Rozée et al. 2020). It embodies profound ethical and socio-economic tensions, with feminist debates revolving around whether it represents coercion driven by poverty or a constrained reproductive choice shaped by systemic inequalities (Donchin 2010; Pande 2014; Saravanan 2016; Rudrappa 2015; Sama Resource Group for Women and Health 2012). While essentialist perspectives emphasize the irreplaceable maternal bond disrupted by market forces, alternative analyses highlight surrogates’ ability to maintain emotional detachment and view their role as work, though some surrogates still express a desire for minimal post-birth contact—such as receiving updates or photos—for reassurance of the child’s well-being, a request that is rarely fulfilled as medical professionals often remove the child immediately after birth to prevent attachment and mitigate potential conflicts with intended parents (Rozée et al. 2020).

While discussions on surrogacy have largely focused on the rights and agency of surrogates, an emerging area of concern is the welfare of children born through these arrangements and recent research has begun to examine whether existing regulations sufficiently protect these children, particularly in cross-border surrogacy arrangements, where legal uncertainties surrounding citizenship, parentage, and identity pose significant risks (NSW Department of Communities and Justice 2024; Wade 2017; Tobin 2014; United Nations 2018; Achmad 2022; Rotabi et al. 2017). Under the CRC, the best interests of children should be a primary consideration (United Nations Committee on the Rights of the Child 2013). However, this principle is tested when citizenship and parentage recognition are denied or delayed, leaving children in legal limbo as many of their fundamental rights depend on legal parenthood and citizenship (Bracken 2024; Rajan 2018; Sifris 2020). In their recent analysis of transnational commercial surrogacy, König and Majumdar (2022) examine how legal conflicts in cases like the Balaz twins—where Germany’s prohibition of surrogacy and India’s refusal to grant citizenship—left the children stateless for an extended period, depriving them of fundamental rights.

The CRC also emphasizes the importance of a family environment for the child’s development. Article 7(1) states: “The child… has as far as possible the right to know and be cared for by his or her parents”. This suggests that a child should have certainty as to who their legal parents are. However, the kinship implications of surrogacy challenge conventional understandings of parenthood and belonging. Surrogacy arrangements inherently disrupt the traditional dyadic model of parenthood, introducing multiple parental figures: intended parents, surrogates, and sometimes gamete donors. The CRC does not define “parents”, creating legal ambiguities in surrogacy cases where competing claims to parenthood arise. Jurisdictions vary in defining legal parenthood—some recognize genetic ties, others prioritize the gestational mother, while some rely on intended parents’ contractual intent—resulting in conflicts and legal uncertainty (König and Majumdar 2022). In this context, legal documentation plays a decisive role in establishing a child’s legal status, citizenship, and familial belonging (König and Majumdar 2022). Birth certificates, passports, and legal decrees do more than grant rights; they construct and legitimize a child’s identity. Yet, as seen in transnational surrogacy disputes, these documents often become sites of legal friction as the law often erases the surrogate mother’s identity, reducing her role to that of a “womb” while reinforcing a single-paternity model through the use of DNA testing and birth certificates (Majumdar 2015; König and Majumdar 2022). Payne (2018) argues that only by legally recognizing more diverse and inclusive forms of reproduction and family formation—where kinship is understood as a multilineal set of relationships between reproductive participants—can we constructively address the inherent tensions between the “kinship grammars” of intent and gestation.

According to the CRC, children have the right to “preserve his or her identity”. Previous research indicates that various practices by clinics—whether deliberately deceptive, or due to inadvertent errors—often render the genetic origins of children born through cross-border surrogacy untraceable (Zannettino et al. 2019). This disparity creates significant challenges for families who seek surrogacy abroad, particularly if parents or donor-conceived individuals later desire access to identifying information about the donor. Additionally, these children may not have the opportunity to learn about or meet their surrogate mother, further complicating their understanding of their origins (Zannettino et al. 2019).

The debate over commercial surrogacy and children’s rights often centres on interpretations of the CRC provisions relating to prohibiting the sale of children (United Nations 2018). A key point of contention lies in whether compensated surrogacy arrangements inherently constitute the sale of children (Tobin 2014). Critics argue that these arrangements involve a transactional process where a child is transferred to intended parents in exchange for monetary or other forms of compensation (Smolin 2015). In contrast, Gerber and O’Byrne (2015) consider that if the purpose of surrogacy is understood to ensure that a child is given to parents who will be able to provide the child with the necessary care and support, commercial surrogacy may not be seen to be the selling of a child.

The interpretation of the rights provided under the CRC by business actors in cross-border surrogacy remains significantly underexplored. Emerging literature has begun to examine the role of surrogacy agents in destination countries, where they act as intermediaries between intended parents, surrogates, and clinics (Deomampo 2016; Nadimpally and Majumdar 2017; Daftuar 2025). However, research has largely overlooked the role of surrogacy facilitators in consumer countries. In destination countries such as India, surrogacy agents operate at the intersection of multiple stakeholders, including pharmaceutical industries, fertility clinics, non-governmental organizations, and international networks, all of which play a role in structuring the surrogacy industry (Deepa et al. 2013; Majumdar 2017). Deomampo (2016, 59) highlights how fertility agencies and medical professionals act as key agents in legitimizing commercial surrogacy, emphasizing that “to reproduce is the strongest human desire, second only to survival itself”. By framing genetic reproduction as a fundamental right, these agents reinforce biogenetic kinship, arguing that “surrogacy is not a crime. It is an alternative mode of delivery for all those who are interested in having their own biological children”. Daftuar (2025) critiques the strategic use of “ethical surrogacy” by Indian surrogacy agencies to legitimize industry interests, often prioritizing fertility clinics and intended parents over surrogates’ rights and well-being. In India’s surrogacy sector, surrogacy agents operate within a stratified system, where those managing and recruiting surrogate mothers—often from lower socio-economic backgrounds, sometimes former surrogates themselves—differ from agents handling financial transactions and legal negotiations with intended parents and clinics, who are typically middle-class professionals with greater access to legal and financial resources (Deomampo 2016; Nadimpally and Majumdar 2017; Majumdar 2017). Thapar-Björkert et al. (2023) find that agents may unintentionally reinforce exploitative structures by maintaining hierarchical relationships with surrogates and upholding industry norms that prioritize intended parents’ interests over surrogate autonomy.

Despite the central role surrogacy facilitators have in shaping industry practices and mediating interactions between key stakeholders, their responsibilities in upholding children’s rights and ethical standards remain inadequately examined. Much of the conversation around children’s rights has been state-centric, focusing primarily on how governments should enforce and implement these rights in cross-border surrogacy (Permanent Bureau 2014; Achmad 2022; Wade 2017). Luo et al. (2022) suggest a “business and human rights perspective” for addressing human rights issues in the surrogacy industry, emphasizing that human right responsibilities must extend beyond the state to include all actors that play a part in these arrangements. This argument stressed, in particular, that market actors are accountable for, and should mitigate against, their direct and adverse impact on human rights. Building on these insights, this paper considers children’s rights as overarching norms that must be respected by all stakeholders, including businesses, involved in cross-border surrogacy. Children’s rights, as enshrined in the CRC, require that they are treated as individuals entitled to dignity, identity, and protection, not simply as commodities in a commercial transaction. However, acknowledging these responsibilities is only part of the solution. The development of policies or arguments focused on what market actors should do is likely to fall short if it is not grounded in an understanding of what market actors actually do and what they consider their responsibilities to be. There are also constraints that market actors face in the context of cross-border surrogacy that complicate their ability to fully uphold children’s rights. As this paper will demonstrate, the legal environment, conflicting interests between intended parents and children, and commercial pressures are closely connected to surrogacy facilitators’ understanding of children as rights holders.

## Situating Cross-Border Surrogacy in Australia

Cross-border reproductive care (CBRC) has become an increasingly prevalent global phenomenon, characterized by individuals crossing national borders to access reproductive treatments (Inhorn and Patrizio 2009; Inhorn and Gurtin 2011). While CBRC expands reproductive opportunities, it also raises significant legal and ethical concerns, particularly regarding the circumvention of domestic regulations (Salama et al. 2018). Many intended parents seek reproductive services abroad due to restrictions or prohibitions in their home countries, a phenomenon that Inhorn and Patrizio (2009) have termed “reproductive exile”. Australia is a key participant in this trend, with many intended parents initially considering domestic surrogacy but ultimately pursuing international arrangements due to the perceived complexity and lengthy processes associated with surrogacy within Australia (Kneebone et al. 2023). Australia’s legal framework permits only altruistic surrogacy, resulting in a limited pool of available surrogates and further incentivizing intended parents to seek services abroad (Sifris et al. 2015; Everingham and Whittaker 2023; Standing Committee on Social Policy and Legal Affairs 2016). Consequently, following the regulatory shutdown of surrogacy markets in countries such as India and Thailand, the USA, Ukraine, and Kenya have become common destinations for Australians seeking surrogacy, offering greater legal certainty, a more structured regulatory environment, and readily available surrogates through professionalized surrogacy providers (Millbank 2015a, b; Kneebone et al. 2023).

While the cross-border commercial surrogacy industry in Australia has been steadily growing, existing legislation fails to effectively address transnational commercial surrogacy activities (Lawson 2022; Jackson et al. 2017). The Commonwealth has no defined role in regulating cross-border commercial surrogacy except through its immigration and citizenship powers. The Department of Home Affairs confers citizenship on the child based on the biological link to the intended parents (Millbank 2013; Stuhmcke 2011). In Australia, the surrogate (birth) mother and her partner are parents at birth, regardless of whether there is any genetic connection to the child (Rajan 2018; Nelson 2013). The Family Court can grant parental responsibility orders to intended parents, allowing them to make significant decisions about a child’s welfare. However, these orders do not confer legal parentage, which is a formal recognition of the parent–child relationship under the law (Australian Law Reform Commission 2024). Although laws in the Australian Capital Territory, Queensland, and New South Wales consider extraterritorial activity illegal, related provisions have never been enforced in any of these jurisdictions, in part because the Family Court has determined that the best interests of the child should outweigh public policy considerations (Stuhmcke 2011).

This ambiguous regulatory landscape has created significant business opportunities for organizations facilitating cross-border commercial surrogacy (Luo et al. 2022). The COVID-19 Pandemic, which began in March 2020, resulted in Australian citizens and permanent residents being barred from leaving the country without a travel exemption. However, despite the border restrictions, it has been reported that the organization Growing Families has helped over a hundred couples obtain travel exemptions to pursue offshore surrogacy arrangements since March 2020 (Fitzsimmons 2021). From an ethical standpoint, this can be seen as an effort to uphold the reproductive autonomy of intended parents, allowing them to pursue parenthood despite unprecedented global mobility constraints. By enabling access to surrogacy arrangements, Growing Families arguably provided crucial support for individuals and couples whose family-building plans might otherwise have been indefinitely delayed. However, questions emerge regarding the extent to which these services align with or potentially challenge Australian surrogacy laws. Additionally, issues arise as to whether such facilitation inadvertently reinforces exploitative practices, particularly in jurisdictions with weak legal protections for surrogate mothers and limited oversight of commercial surrogacy arrangements (Luo et al. 2022).

The involvement of Australian medical professionals in assisting intended parents with cross-border surrogacy presents significant ethical and legal challenges (Millbank 2019; Zannettino et al. 2019). The National Health and Medical Research Council’s (NHMRC) Ethical Guidelines explicitly discourage clinicians from engaging with or facilitating commercial surrogacy arrangements, stating:

It is ethically unacceptable to undertake or facilitate surrogate pregnancy for commercial purposes. Clinics must not undertake or facilitate commercial surrogacy arrangements (Millbank 2015a, b).

As a result, medical professionals often find themselves in a difficult position, balancing their ethical obligations with the needs of patients seeking surrogacy options abroad (Millbank 2019). Medical professionals, aware of the legal risks associated with providing information about surrogacy, often adopt a neutral stance or limit their discussions to overseas surrogacy and its potential challenges (Zannettino et al. 2019). This cautious approach restricts the ability of healthcare providers to offer comprehensive care and guidance, which, in turn, reduces transparency and hampers informed decision-making for intended parents (Zannettino et al. 2019). Intended parents, lacking adequate professional support, often resort to online platforms or surrogacy events for information (Zannettino et al. 2019; Luo et al. 2023). These events frame intended parents as consumers and promote the perceived benefits of international surrogacy while downplaying the ethical, legal, and social complexities (Luo et al. 2023).

Adding to the complexity is the absence of clear legal guidelines addressing the roles and responsibilities of other surrogacy facilitators—such as legal advisors, surrogacy agencies, and counselors—especially in the cross-border context. Notably, the term “facilitation” has never been explicitly defined, leaving considerable ambiguity regarding what constitutes prohibited practices (Millbank 2019). This regulatory gap introduces a host of ethical and legal challenges as facilitators navigate the tensions between market demands, professional obligations, and the risks posed to surrogates and children (Millbank 2018; Luo et al. 2022). Moreover, the absence of standardized protocols leads to considerable variation in the quality of care and support provided to all parties involved (Shenfield et al. 2011). A stark example of these challenges occurred in 2023 when several Australian intended parents participating in international commercial surrogacy arrangements found themselves unable to access embryos and other genetic material. This followed the sudden closure of a fertility clinic in Greece, which faced allegations of human trafficking, record falsification, and mistreatment of surrogate mothers. Significantly, Australian industry professionals reported no prior indications of irregularities in the clinic’s operations, underscoring the limitations of due diligence and oversight in international surrogacy arrangements (Luo et al. 2022; NSW Department of Communities and Justice 2024). It is within this context of growing cross-border commercial surrogacy, regulatory uncertainties, and practical challenges that this paper examines the perspectives of facilitators on the rights of children born through such arrangements.

## Methodology of the Study

This section outlines the methodology used in this research and its limitations, emphasizing that this study serves as an initial exploration of the perspectives of surrogacy facilitators on children’s rights within cross-border commercial surrogacy arrangements. The findings presented are based on interviews with surrogacy facilitators and are intended to provide a foundation for future, more extensive studies. The research was approved by the Human Research Ethics Committee (Ethics Approval granted in 2019, research ethics identification number: 21720) prior to the commencement of data collection.

Seven surrogacy facilitators from Victoria were interviewed using a semi-structured, in-depth approach between October 2019 and March 2020. The participants included four counsellors, three lawyers, and a consumer advocate, all of whom primarily work with intended parents. These facilitators had extensive professional experience in the field, ranging from ten years to over forty years. For example, one lawyer had been involved in surrogacy cases for four decades. Victoria was selected as the focus for this research because cross-border commercial surrogacy is not explicitly prohibited in the state, making it easier to approach surrogacy facilitators compared to other Australian states where such arrangements are criminalized.

Due to the small number of participants in this study, specific measures have been taken to mitigate the risk of identification and ensure confidentiality. Personal information, such as participants’ specific roles, professional experience, or unique case details, is only mentioned when directly relevant to the analysis or discussion. In all other instances, participants are referred to collectively as “facilitators” to maintain a general sense of anonymity. This approach minimizes the potential for individual participants to be identified, especially given the close-knit nature of the surrogacy facilitation field in Victoria.

It is also important to emphasize that the study does not seek to generalize its findings to the specific roles of counselors, lawyers, or consumer advocates. While the participants’ professional backgrounds provide valuable context, the study’s primary focus is on understanding facilitators’ perspectives as a group. The insights presented here aim to offer a broad overview of how facilitators perceive and navigate issues related to children’s rights in cross-border surrogacy arrangements. The intent is not to compare or draw conclusions about the practices of different professional roles but to highlight the collective experiences and viewpoints that emerged from the interviews.

This methodological approach allows the study to focus on exploring the facilitators’ shared perspectives and the challenges they encounter while providing a foundation for future research. A larger-scale study with a more diverse and representative sample could build upon these findings to analyze the nuances between specific roles and their contributions to the surrogacy process in greater detail.

Thematic analysis was conducted to identify and develop key themes from the data, using the inductive approach described by Braun and Clarke (2012). Interview transcripts were imported into NVivo11™, a qualitative data management software, to facilitate the analysis process. Perspectives from participants were grouped into themes, allowing for a structured understanding of their views and practices.

While this study provides valuable insights into the human rights dimensions of surrogacy facilitation, several limitations must be acknowledged. The research was conducted solely in Victoria, meaning the findings may not be generalizable to other states or countries with different regulatory frameworks. Additionally, the self-selecting nature of the participants—most of whom identified as ethical or “good” market players—may have introduced bias, as their perspectives may not fully represent the broader practices of surrogacy facilitators. Future research could address these limitations by expanding the sample size, including participants from other jurisdictions, and employing additional methods such as surveys and focus groups. These steps would enable more robust and comprehensive insights into the role of surrogacy facilitators in navigating human rights issues in cross-border surrogacy.

## Findings

This section presents the findings from interviews conducted with various facilitators involved in cross-border surrogacy arrangements. When analyzing the interviews, it became evident that facilitators generally acknowledged the importance of recognizing children as holders of rights. However, their discussions frequently framed these rights as requiring a delicate balance with competing considerations. The findings are organized into four main themes: child-centric responsibility, advocates for the child’s identity rights, structural limitations in combating commodification in cross-border surrogacy, and legal barriers to protecting children’s rights.

### Child-Centric Responsibility

In this study, surrogacy facilitators were asked about their general understanding of children’s rights in the context of cross-border surrogacy. Their responses reflected a strong alignment with the principles of the CRC, particularly the paramount importance of prioritizing the best interests of the child in all decisions and actions. One facilitator highlighted her commitment to the child’s well-being, stating, “The best interest of the child is the paramount thing that I think about”. She further advocated for recognizing the child as an individual with inherent rights, emphasizing, “Children have their own rights”. Her role, as she sees it, is to ensure intended parents understand their ethical responsibilities—not only in bringing a child into the world but also in protecting the child’s fundamental rights from the earliest stages of surrogacy planning.

A lawyer stressed the professional duty to prioritize the child’s best interests. He explained:

As lawyers, we have that responsibility; it’s hard coming from [a family law] background to deviate from the idea of the child’s best interests as the paramount consideration.

He noted that his extensive experience shapes his approach to surrogacy cases:

I generally approach all matters from the perspective of how this will impact a child who might be born through an arrangement. How are their interests being protected and catered to?

For him, a child-centric perspective is fundamental to professional practice. Several facilitators emphasized their responsibility to educate intended parents about the importance of prioritizing the child’s rights, acknowledging that the intense desire for a child can sometimes overshadow considerations for the child’s welfare, particularly in cross-border surrogacy arrangements. One facilitator observed that some clients express sentiments like:

We just want the baby… We’re not really thinking about what’s best for this child.

He further explained,

When you look at who intended parents are, they are desperate to have children. Desperate people don’t always think rationally.

Another facilitator identified a significant challenge for intended parents in recognizing the child as an individual with their own rights, feelings, and future needs. She explained:

People need to be making informed decisions that are really hard to make when you’re thinking about a baby that doesn’t exist yet. It’s that baby lust. We really want the baby, but they haven’t actually thought about what that baby will think in 18 or 25 years, or when they’re having their own children.

She emphasized the difficulty of envisioning a child’s future perspective:

Most of us don’t have a concept of what that looks like, and it can be hard to think about what that child might say in the future. But we need to focus on the child’s best interests. When people tell me, ‘We just want the baby, and we don’t care who the donor is, as long as she’s beautiful, as long as the surrogate does what she’s told and doesn’t eat soft cheese,’ I think, ‘We’re not really considering what’s best for this child.’

The interviews provided detailed examples of how facilitators adopt a proactive approach to addressing these concerns, emphasizing the importance of thoughtful, informed decision-making over immediate desires or “the urge to have a baby”. One facilitator explained that a key part of her role is preparing intended parents for difficult possibilities, such as the birth of a child with deformities or disabilities. She stressed the importance of discussing these possibilities at the very beginning of the surrogacy process, ensuring that all parties involved are aligned on how to handle such situations.

Some facilitators interviewed emphasized their critical role in ensuring that intended parents engage with surrogacy with a clear and realistic understanding of their responsibilities. One facilitator shared examples where intended parents were unable to reach agreement on key issues, such as their willingness to parent a child with unexpected medical needs. In these instances, she explained, she might recommend postponing or reconsidering the surrogacy process altogether, recognizing that unresolved disagreements could result in harmful consequences for the child.

According to the interview, parental decisions during surrogacy planning shape the extent to which the child’s rights are respected and protected. Facilitators, therefore, play a vital role in guiding intended parents to align their decisions with a rights-based framework, ensuring that the child’s welfare remains central throughout the cross-border surrogacy process.

### Advocates for the Child’s Identity Rights

Interviews with facilitators in cross-border surrogacy arrangements highlighted the critical importance of ensuring that children born through cross-border surrogacy arrangements have access to information about their genetic and gestational origins. They perceived their roles as not only professional advisors but also as advocates for the child’s right to identity. One facilitator emphasized the necessity of transparency regarding the child’s genetic and gestational origins for the rights of children born through surrogacy. This facilitator stated, “The most important thing in terms of rights for children is full disclosure from their parents of the way they were created”. This facilitator saw his role as encouraging openness between parents and children, believing that transparency about the child’s genetic and gestational origins supports the child’s identity development and aligns with ethical practices that respect their rights.

Similarly, another facilitator highlighted the significance of children having access to information about their genetic and gestational origins, underscoring their role in guiding intended parents toward honesty. This facilitator remarked:

It’s important for children to have access to information about their donors and their surrogates so that they can work out their own identity around that. Knowledge is much better than pretending or keeping it a secret.

This facilitator viewed her professional practice as a responsibility to counsel parents on the long-term benefits of transparency regarding the child’s genetic and gestational origins for the child’s sense of self, emphasizing that openness is crucial for fostering a secure and coherent identity:

I don’t think that’s right for anyone… when we’re telling these stories to our children, do we think it’s a good story to tell? Is there anything that we’re uncomfortable about telling? If we’re saying to our children as they get older, ‘You had a surrogate, we don’t remember her. We don’t have contact with her. Or we had an egg donor, but we don’t know her name.’ I think that’s something that we need to be careful of. These kids are growing up and saying, ‘That’s not what I want. I want to know who my donor was. I want, not just medical records, but I want to know her name. I want to know what she looked like.’

This facilitator’s stance illustrates how the facilitator actively discourages anonymity in surrogacy arrangements, as it could infringe on the child’s right to know their origins. The facilitator sees it as a duty to prevent the creation of arrangements where the child’s right to this knowledge is compromised.

### Structural Limitations in Combating Commodification in Cross-Border Surrogacy

Facilitators interviewed in this study expressed significant concerns about the commodification of children in commercial surrogacy, particularly in cross-border arrangements. One facilitator emphasized the transactional nature of surrogacy and its potential to commodify children:

I think it’s really recognizing that they [intended parents] see the baby as property, it’s a commodity that they’ve decided they don’t want to have anymore and then just going to discard it.

Facilitators also pointed out that cross-border surrogacy arrangements are often structured to guarantee that intended parents receive a baby. One facilitator noted that in some overseas surrogacy settings, intended parents are assured by agencies or lawyers that they will receive the baby, even if the surrogate wishes to keep the child:

The overseas ones will also talk about, essentially that if she decided to keep the baby, that the agency or the lawyers over there will enforce the contract against her and they’ll get the baby and give it to the intended parents. Now, I don’t know how that works, but I do know it’s very much that the intended parents are told, no matter what happens, they’ll get the baby at the end, and that it’s their baby, it’s their property, I guess… it’s probably the right phrase. That’s why they’re treated. They’re paying all this money, they’re signing contracts, they’ll get the baby at the end. As a surrogate, I find that really upsetting, that she’s treated like a commodity…

The contractual guarantees given to intended parents reflect a property-based view of the arrangement, where their rights to the baby are prioritized due to their financial investment. A lawyer who frequently reviews international surrogacy contracts explained:

The selling point of many surrogacy contracts is ensuring that intended parents receive a baby at the end of the pregnancy…. In reviewing overseas contracts, I often see that they are heavily weighted in favor of the intended parents, focusing primarily on managing the surrogate’s behavior to ensure the intended parents ultimately receive a child…. It’s all about managing the surrogate’s behavior and conduct and really about making sure that the intended parents get a baby at the end of it. That’s the way they write them.

According to this interviewee, despite strong personal advocacy against commodifying children, the inherent power imbalances and restrictive clauses within these contracts make it challenging to address such ethical concerns fully. These limitations reflect broader systemic issues within the surrogacy industry, where economic considerations often overshadow the need to uphold the rights and dignity of the child and the surrogate.

### Legal Barriers to Protecting Children’s Rights

Facilitators interviewed for this research identified significant legal barriers to protecting children’s rights in cross-border surrogacy arrangements. One of the primary challenges highlighted was the lack of alignment between state and federal laws governing surrogacy and parentage recognition. Surrogacy arrangements fall under state and territory jurisdiction, while the determination of parentage is regulated federally by the Family Law Act. This fragmentation complicates the process of establishing legal parentage, particularly for children born overseas. One facilitator emphasized,

There’s a problem at the moment with the Family Law Act, and the difference between the Family Law Act and the state act—you can’t get a parentage order if you’ve done surrogacy overseas.

As a result, according to the facilitators, many intended parents are unable to obtain a parentage order, leaving their child without secure legal recognition, which contradicts the best interests of the child and leaves them vulnerable to legal uncertainties. A facilitator criticized the Australian legal system for failing to recognize the legal parentage of intended parents in cross-border surrogacy cases, arguing that this violates the best interests of the child:

We’ve also got an issue here in Australia where those families who use overseas surrogates are not recognized as the legal parents of that child. Under human rights guidelines, it’s difficult to see that as in the best interest of the child to have no legal parent living with them.

However, the interviews revealed that granting intended parents legal parentage in cross-border surrogacy cases does not always align with the best interests of the child. Facilitators highlighted instances where intended parents reconsider their decision, particularly if their personal circumstances change during the waiting period. One facilitator described the risks involved:

The risk is actually greater, quite significantly greater, that the surrogate will end up raising a child that she didn’t want than it is for the intended parents to walk away without a child they did want. They walk away because they’ve decided they don’t want the baby, and it might be because they’ve separated and decided they don’t want to collect the baby at the end.

To address this issue, facilitators noted the lack of standardized background checks or psychological evaluations in many cross-border surrogacy arrangements. In response, they have taken on the informal responsibility of assessing the mental and emotional preparedness of intended parents. A counselor described her approach:

I’ll also go into their psychological history, look at their mental health history. Are there any risks to them in their mental health in terms of how they're going to cope in becoming a parent? And particularly long distance, where they haven’t got the connection to that pregnancy because they can only go overseas maybe for two or three times during that pregnancy for key ultrasounds.

The interviews also highlighted examples of situations where practical recognition of intended parents’ parental status is achieved through informal means, even in jurisdictions where formal legal recognition may be absent or where surrogacy arrangements are legally contentious. One interviewee pointed out that in certain jurisdictions, the issuance of a birth certificate naming the intended parents can provide sufficient parental status for managing everyday responsibilities. As the interviewee explained:

If you live in a jurisdiction where procuring a child through offshore surrogacy is against the law, then the last thing you’re going to do is bring yourselves to the attention of the court… in a day-to-day sense, they don’t need to get it because, in theory, you have parental responsibility which has been given to you from the birth certificate… In reality, when you want to enrol your child or take them to a day-care centre or school… no one’s interested in whether you’ve got parental responsibility in the formal sense.

While practical recognition through birth certificates allows intended parents to navigate daily responsibilities without legal scrutiny, it often comes at the expense of formal legal security. This trade-off may leave both the child and intended parents vulnerable, particularly in situations involving custody disputes and inheritance rights.

## Concluding Discussion

The role of surrogacy facilitators varies across jurisdictions, shaped by distinct legal frameworks and the structural positioning of consumer and destination countries within the global reproductive industry network (Luo et al. 2022; Luo et al. 2023; Vertommen et al. 2022). In Australia, where commercial surrogacy is strictly prohibited but cross-border surrogacy remains a legal grey area, facilitators operate within a consumer country model. Unlike facilitators in commercial surrogacy markets who manage surrogates directly, Australian facilitators, according to the interviews, primarily serve as advisors, ensuring that intended parents understand their responsibilities, particularly in cross-border arrangements where legal parentage, citizenship, and the child’s best interests can become complex and contested (Deomampo 2016; Nadimpally and Majumdar 2017; Majumdar 2017).

This study examines how the views and practices of surrogacy facilitators involved in cross-border surrogacy in Australia align with the principle of children as autonomous holders of rights. Children’s rights, broadly outlined in the CRC, are assessed in relation to their recognition and application within their practices. These rights are evident in facilitators’ approaches, particularly in their emphasis on safeguarding the welfare of the child and prioritizing the child’s best interests throughout the surrogacy process. For instance, facilitators acknowledged children’s rights to identity, family relationships, and access to information about their genetic and gestational origins. Notably, facilitators in Australia emphasize that safeguarding the child’s best interests requires acknowledging the interconnectedness of the surrogate’s role, the donor’s identity, and the child’s right. Unlike Indian surrogacy agents, which frames surrogacy as a transaction that ends at birth and discourages contact with surrogate mothers, Australian facilitators recognize that ongoing social bonds play a crucial role in shaping a child’s sense of belonging (Rozée et al. 2020).

However, the study reveals persistent tensions. Cross-border surrogacy arrangements are frequently embedded in market-driven, profit-oriented contexts (United Nations 2018; Fronek 2018). While surrogacy facilitators in Australia often express alignment with rights-based principles, their daily practices frequently necessitate navigating competing priorities. A central theme both in the literature and in the findings is the tension between the right of intended parents to have children and the rights of the child in surrogacy arrangements (Achmad 2022). According to the interviews, while intended parents seek to realize their desire for parenthood, this pursuit can sometimes overshadow the child’s best interests. Facilitators strive to mediate this tension by advocating for the child’s best interests and ensuring that intended parents are aware of the rights of the child. This study also shows that facilitators face significant structural limitations in combating the commodification of children in cross-border surrogacy. The interviews revealed that surrogacy contracts are often heavily skewed in favor of intended parents, treating children as commodities to be delivered at the end of the process. While previous research suggests that market actors trust the market to “find its own level”, interviews in this study reveal that facilitators believe market pressures frequently create conditions where children’s rights are overshadowed by the goals of intended parents (Millbank 2018).

Scholars such as König and Majumdar (2022) and Sifris (2020) note that jurisdictional conflicts in defining legal parentage and legal prohibitions on commercial surrogacy frequently leave children in precarious legal limbo. This concern is also highlighted by surrogacy facilitators in interviews, who point to the misalignment between state and federal laws governing surrogacy and parentage in Australia. This discrepancy not only creates confusion but can also compromise the best interests of the child as many of their rights are secured through legal parenthood. As intended parents often struggle to secure formal legal recognition—especially when children are born through overseas surrogacy—concerns arise regarding potential legal uncertainty. During the interviews, while some facilitators advocated for granting parentage to intended parents, others emphasized that merely granting legal recognition to intended parents does not automatically safeguard the child’s best interests. Facilitators highlight that intended parents may change their minds about raising a child, particularly in cases where their personal circumstances shift before the child’s birth. This perspective reinforces the idea that a child’s best interests should not be limited to recognizing only the intended parents as legal parents but should instead consider kinship as a network of relationships among reproductive parties that can take multilineal forms (Payne 2018). Facilitators note that, in jurisdictions where formal parentage orders are difficult to obtain, intended parents can rely on informal mechanisms, such as birth certificates, to function as legal parents in daily life. This phenomenon underscores a broader disconnect between legal formalities and social practices in transnational surrogacy, as highlighted by König and Majumdar (2022). The birth certificate, in this context, becomes a crucial legal workaround, enabling intended parents to function as legal guardians through the implicit acceptance of administrative systems rather than through formal legal recognition. However, the use of birth certificates as a substitute for parentage orders can generate legal friction, as it misrepresents the reality of cross-border surrogacy by listing only the intended parents as the child’s legal parents. This erasure disregards the complex kinship structures at play, where children may have multiple parental figures—intended parents, surrogate mothers, and gamete donors. By omitting details of the surrogate mother and gamete donor, the child’s legal identity is rewritten exclusively through the framework of intended parenthood, marginalizing the surrogate mother’s role despite her significant contribution to the surrogacy process. Majumdar (2015) critiques this practice, arguing that it reduces the surrogate mother to a mere “womb” by using DNA testing and birth certificates to establish parentage.

### Implications

In light of the Albanese Government’s 2024 request for the ALRC to review Australia’s surrogacy laws, it is clear that strengthening protections for children’s rights is both timely and necessary (Australian Law Reform Commission 2024). From the interviews with surrogacy facilitators, it is evident that while their assistance can help intended parents to fulfill their reproductive aspirations, it also exposes significant gaps in Australia’s surrogacy laws—particularly the lack of a clear and consistent framework for recognizing parentage in international surrogacy cases. Judge Alexandra (2021) of the Federal Circuit Court of Australia noted that the recognition of legal parenthood extends beyond legal entitlements, playing a crucial role in a child’s sense of identity and belonging throughout their life, and suggested that Australia must reform its existing laws to truly protect the rights of children born through surrogacy. A critical aspect of reform should involve the implementation of rigorous screening and assessment processes for intended parents. Facilitators emphasize that the lack of standardized background checks and psychological evaluations increases the risk of intended parents reconsidering their decision, particularly if personal circumstances change during the surrogacy process. Establishing pre-surrogacy assessments as a prerequisite for legal parentage would ensure that only those who meet clearly defined criteria are granted parental recognition, reducing the reliance on informal mechanisms and mitigating risks associated with parental abandonment or legal uncertainty for the child. Additionally, the rigid application of the two-parent model in Australian law fails to account for the complex kinship structures inherent in cross-border surrogacy, where multiple parties—including the intended parents, the surrogate, and, in some cases, gamete donors—may have significant roles in the child’s life (Gerber and Lindner 2015). This underscores the need for a re-evaluation of parentage laws to allow for more flexible, multilineal kinship structures that recognize the unique nature of surrogacy arrangements.

In advocating for children’s rights, this study aligns with the critical perspective of Luo et al. (2022), emphasizing that market actors should take greater responsibility in protecting human rights. Effectively realizing rights in cross-border surrogacy practices necessitates the active engagement of business actors alongside state interventions (Luo et al. 2022). Relying solely on state mechanisms has its limitations, as states often lack the capacity or jurisdictional reach to fully regulate cross-border arrangements. This study’s findings emphasize that surrogacy facilitators’ interpretations of children’s rights and their professional roles significantly influence surrogacy practices. Strengthening a children’s rights perspective in this context necessitates a focused effort to enhance facilitators’ legal awareness and accountability, particularly in light of Australia’s ambiguous and inconsistent regulatory framework. Certification of surrogacy services presents a compelling solution, as highlighted by scholars (Luo et al. 2022; Luo 2024a, b) and recent advancements in the UK surrogacy law. In 2023, the Law Commissions of England and Wales and Scotland published a comprehensive review of surrogacy laws, proposing the licensing and regulation of non-profit surrogacy organizations through the Human Fertilisation and Embryology Authority (HFEA). Though the UK model focuses on non-profit entities, its core rationale rests not only on the financial structure per se, but on fostering accountable and ethically compliant facilitation through structured oversight and transparent standards. In adapting this logic to Australia, regulators might establish a certification scheme requiring surrogacy facilitators to demonstrate advanced competency in children’s rights, developmental psychology, cultural sensitivity, and the ethical principles inherent in informed consent, while ensuring that intended parents receive substantive, child-centred counselling at the outset. A key element of this approach would be the creation of a formal accreditation mechanism overseen by an independent authority that articulates and enforces ethical standards, provides regular monitoring, and offers accessible complaint procedures. By implementing a certification system that emphasizes ethical accountability and child-centred practices, Australia can move toward a surrogacy framework that consistently upholds the rights and welfare of children born through these arrangements.

From a global perspective, scholars underscore the complexity of regulating cross-border surrogacy, noting the need for in-depth empirical research and thoughtful normative deliberations (Zafran and Hacker 2019). According to interview findings, the realization of children’s rights is closely intertwined with the need for effective global regulation. The surrogacy market has become increasingly globalised; however, there is no corresponding global regulation in place, despite attempts to establish an international treaty. Nevertheless, creating universal rules is challenging due to the significant disparity between states (Tobin 2014). It is suggested that the regulation of the surrogacy industry should depend on a “repro-regional moral framework” that considers local societal values, economic, and political aspects (Smietana et al. 2021). While the creation of universal standards for international surrogacy businesses is complicated by widespread moral variations, establishing a regional regulatory mechanism to bridge these differences remains feasible. Such an approach would require close collaboration between market actors, government authorities, and industry stakeholders, all working collectively to uphold ethical standards and protect everyone involved—particularly children (Luo 2024a, b). Countries with similar moral and ethical commitments around surrogacy could thus form partnerships to develop coherent, stable regulatory standards. Within these regions, businesses with comparable ethical principles may be more inclined to coordinate their practices. Such cooperation could involve crafting shared guidelines designed to safeguard children’s rights and ensure consistent standards for critical regulatory elements—ranging from rigorous background checks on intended parents to clear, standardized mechanisms for determining legal parentage. Ultimately, such an approach helps ensure that children’s best interests remain at the forefront of policy and practice.

## Data Availability

The data supporting this study are not publicly available due to confidentiality and privacy concerns.
